# Validation and comparison of 2D grading scales and 3D volumetric measurements for outcome assessment of bone-grafted alveolar clefts in children

**DOI:** 10.1093/ejo/cjae002

**Published:** 2024-02-12

**Authors:** Mathias Lemberger, Daniel Benchimol, Marie Pegelow, Reinhilde Jacobs, Agneta Karsten

**Affiliations:** Division of Orthodontics, Department of Dental Medicine, Karolinska Institutet, Box 4064, 141 04 Huddinge, Sweden; Eastman Institute, Department of Orthodontics, Public Dental Services Stockholm, Box 6031, SE-102 31, Stockholm, Sweden; Division of Oral Diagnostics and Rehabilitation, Department of Dental Medicine, Karolinska Institutet, Box 4064, 141 04 Huddinge, Sweden; Division of Orthodontics, Department of Dental Medicine, Karolinska Institutet, Box 4064, 141 04 Huddinge, Sweden; Division of Oral Diagnostics and Rehabilitation, Department of Dental Medicine, Karolinska Institutet, Box 4064, 141 04 Huddinge, Sweden; OMFS IMPATH Research Group, Department of Imaging & Pathology, Faculty of Medicine, University of Leuven and Oral & Maxillofacial Surgery, University Hospitals Leuven, Campus Sint-Rafaël |Kapucijnenvoer 33, BE-3000 Leuven, Belgium; Division of Orthodontics, Department of Dental Medicine, Karolinska Institutet, Box 4064, 141 04 Huddinge, Sweden

**Keywords:** alveolar bone grafting, cleft lip and palate, CBCT, cone beam computed tomography, treatment outcomes, indices for alveolar bone grafting

## Abstract

**Background:**

Several methods have been proposed to assess outcome of bone-grafted alveolar clefts on cone beam computed tomography (CBCT), but so far these methods have not been compared and clinically validated.

**Objectives:**

To validate and compare methods for outcome assessment of bone-grafted clefts with CBCT and provide recommendations for follow-up.

**Methods:**

In this observational follow-up study, two grading scales (Suomalainen; Liu) and the volumetric bone fill (BF) were used to assess the outcome of 23 autogenous bone-grafted unilateral alveolar clefts. The mean age at bone grafting was 9 years. The volumetric BF was assessed in five vertical sections. The bone-grafted cleft outcome was based on a binary coding (success or regraft) on a clinical multidisciplinary expert consensus meeting. Grading scales and volumetric assessment were compared in relation to the bone-grafted cleft outcome (success or regraft). Reliability for the different outcome variables was analyzed with intra-class correlation and by calculating kappa values.

**Limitations:**

The study had a limited sample size. Clinical CBCT acquisitions had a varying tube current and exposure time.

**Results:**

Volumetric 3D measurements allowed for outcome assessment of bone-grafted alveolar clefts with high reliability and validity. The two grading scales showed highly reliable outcomes, yet the validity was high for the Suomalainen grading scale but low for the Liu grading scale.

**Conclusions:**

Volumetric 3D measurement as well as the Suomalainen grading can be recommended for outcome assessment of the bone-grafted cleft. Yet, one must always make a patient-specific assessment if there is a need to regraft.

## Introduction

Orofacial clefts are congenital malformations arising when facial prominences fail to unite during early pregnancy. The malformation is common with an incidence of approximately 1/730 live births annually [1]. If the cleft involves the alveolus, bone grafting is mandatory. Most cleft centers perform bone grafting in the mixed dentition to unify the maxilla and to provide bone support for the nose and the erupting permanent teeth [2, 3]. A successful bone graft stimulates bone ingrowth into the alveolar cleft and is essential for the development of good periodontal tooth support.

The use of cone beam computed tomography (CBCT) for evaluation of the bone-grafted cleft alveolus is increasing as it permits three-dimensional (3D) assessment of the bone crest with lower radiation doses than conventional multislice CT scans [4–14]. Since 2016, the cleft team in Stockholm uses CBCT routinely to evaluate the bone-grafted alveolar cleft. The CBCT scans are assessed by a multidisciplinary team for a consensus if there is enough bone fill (BF) in the alveolar cleft or if the patient needs a regraft for the future rehabilitation of the cleft. The multidisciplinary team consists of radiologists, plastic surgeons, and orthodontists.

In several previous studies, the bone-grafted alveolar cleft has been evaluated by volumetric assessment of the BF on CBCT scans [15–20]. Effort has also been made to develop grading scales to assess the bone-grafted cleft alveolus [4, 6, 12–14]. The grading scales assesses bone grafting by two-dimensional (2D) measurements at different height levels to provide 3D assessment. The grading scale presented by Soumalainen *et al.* [6] evaluates the bone support of the bone-grafted cleft at three vertical dental levels. In addition, the horizontal level of the nasal floor at the cleft side is compared with the nasal floor level at the non-cleft side. The grading scale presented by Liu *et al.* [4] assesses the interdental septal thickness in the bone-grafted alveolar cleft at three vertical dental levels,

Volumetric assessment of the BF for the total cleft can be a difficult measure to use in a clinical situation as it does not provide information regarding spatial location of the bone graft. In the present study, the alveolar cleft was divided into five vertical levels to overcome this problem. To our knowledge, there is no previous study comparing the validity of volumetric assessment and grading scales [4, 6] for analyzing outcome after bone grafting to the alveolar cleft on CBCT scans. Therefore, the aim of this study was to assess and validate 3D volumetric measurements of the grafted alveolar cleft and compare the outcome measures to those obtained by 2D grading using the Soumalainen and Liu grading scales [4, 6].

The null hypothesis was that there is no difference in the assessment of success or regraft after bone grafting the alveolar cleft when comparing two different grading scales and 3D volumetric assessments on CBCT scans.

## Subjects and methods

### Patient selection

Consecutive records including CBCT scans from children born with a unilateral alveolar cleft who underwent reconstruction of the alveolar cleft with a bone graft from the iliac crest at the Stockholm cleft team were collected. The CBCT scans were taken between 2016 and 2017, one CBCT scan for planning, and one CBCT scan for evaluating the bone graft. Records from twenty-nine (*n* = 29) patients were initially included in the study. Children planned for a second bone graft (*n* = 3) as well as patients with scans that had presence of image artifacts (*n* = 3) were excluded. The final number of included participants was 23 (19 males, 4 females: mean age 9 years, and 3 months with a standard deviation of 1 year and a range of 7 years and 10 months to 10 years and 11 months). Sixty-five percent had the cleft on the left side, while the remaining 35% had the cleft on the right side.

### Surgical procedure

The secondary alveolar bone grafting (SABG) procedure was performed by grafting the alveolar cleft with cancellous bone harvested from the iliac crest according to the method introduced by Boyne and Sands [2, 3].

### Image acquisition

CBCT examinations were performed in the mean of 5.8 months before SABG and in the mean of 6.6 months after SABG with a Promax 3D Mid scanner (Planmeca OY, Helsinki, Finland). The image conditions chosen for the examination were 90 kV tube voltage, 4.5–8 mA tube current, 4 s or 12 s exposure time, voxel size 0.2 mm, and a field of view of 80 mm in diameter and 50 mm in height. For the evaluation, axial and coronal reconstructions of 0.25 mm thick slices were made with the software program Romexis (version 5.1) (Planmeca OY, Helsinki, Finland). Data were saved and stored as Digital Imaging and Communications in Medicine files (DICOM) files.

### Volumetric measurements for 3D assessment

**Figure 1.**
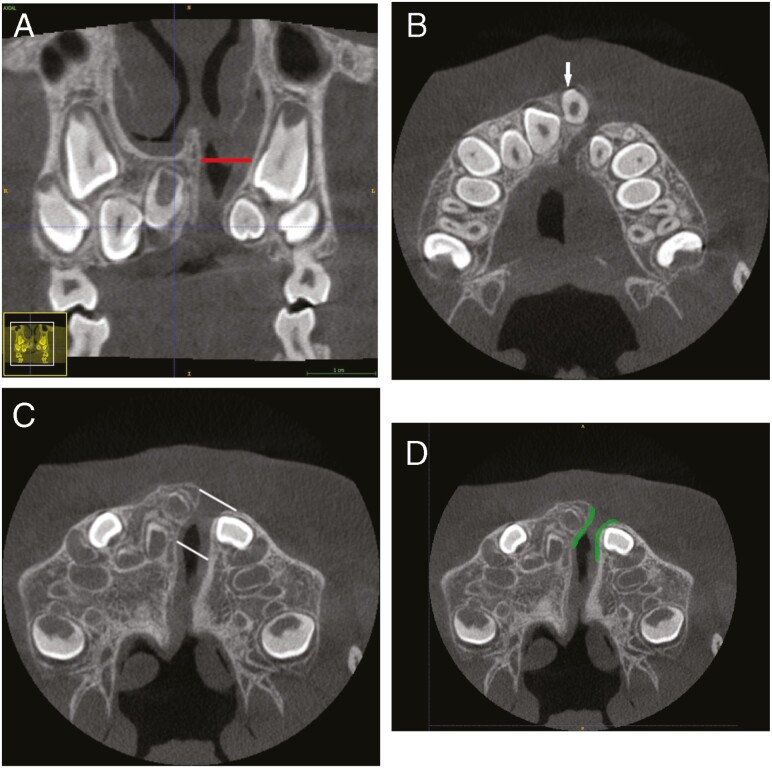
Anatomical landmarks used as guidelines to define the boundaries of the cleft prior to segmentation. (A) Coronal plane illustrating the superior boundary toward the nasal cavity (red line). (B) Axial plane illustrating the inferior boundary defined by the most superior part of the cemento–enamel junction (arrow) of the central incisor on the cleft side. (C) Axial plane illustrating the buccal and palatinal boundary (white lines) defined by drawing straight lines from the medial and lateral segments and by the guidance of the incisive foramen. (D) Axial plane illustrating how the mesial and the distal boundaries of the cleft were defined semi-automatically with the adaptive brush tool in the software ITK-SNAP (green linings).

**Figure 2.**
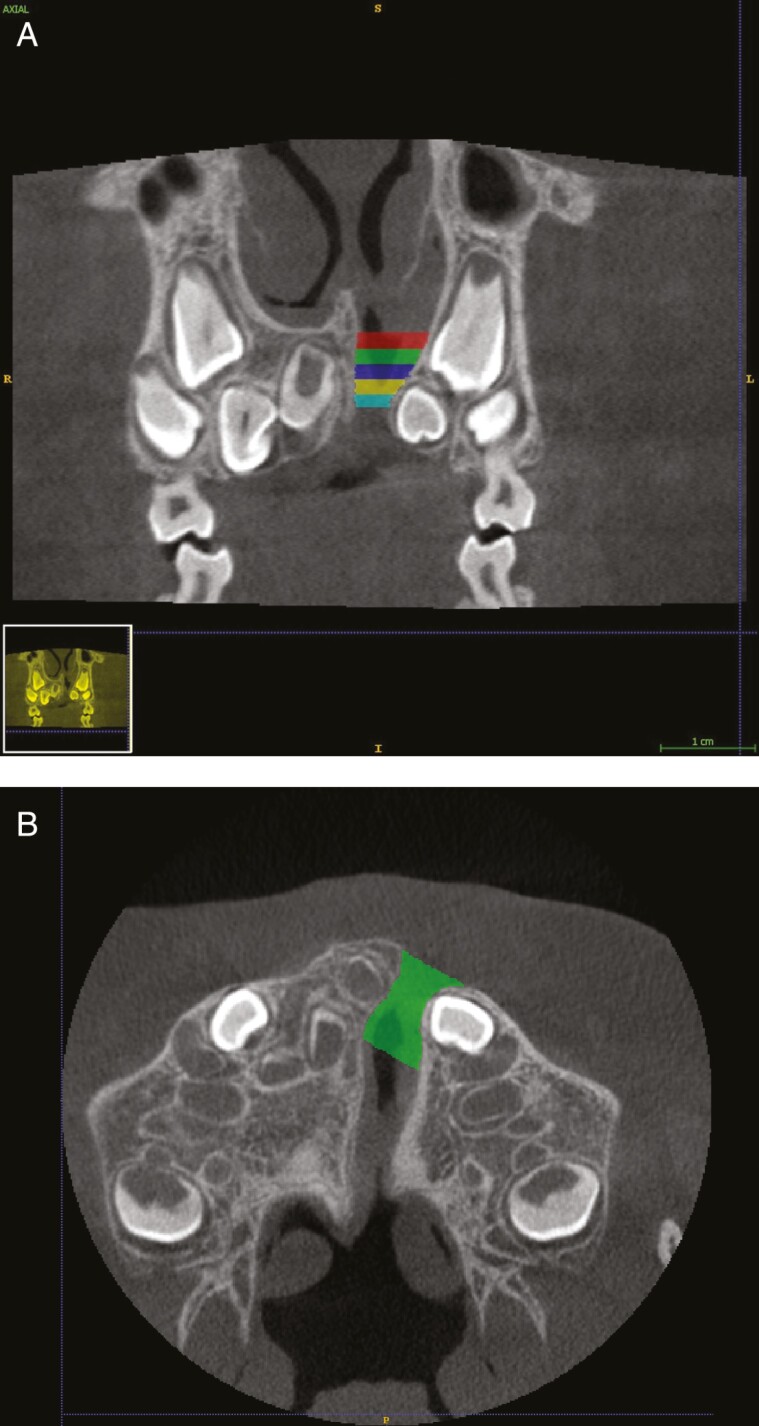
(A) Coronal plane illustrating how the cleft was vertically divided into five sections. One N section (red) and four dental sections A (green), MA (blue), MC (yellow), and cervical (C, turquoise). (B) Axial plane illustrating segmentation in the dental apical section of the cleft (green).

For volumetric measurements, the DICOM files from the CBCT examination were imported to a segmentation software, ITK-SNAP (version 3.6.0; Paul Yushkevich, Ph.D., University of Pennsylvania and Guido Gerig, Ph.D., University of Utah, USA). The anatomical boundaries of the alveolar cleft were assessed using anatomical landmarks as guidelines manually and semi-automatically with help of the adaptive brush tool in the software ITK-SNAP (Fig. 1a–d). Before performing anatomical landmarks identification and segmentations of the anatomical boundaries, observers were trained and calibrated based on a protocol established by a multidisciplinary expert consensus group. The cleft was vertically divided into one N section and four dental sections (Fig. 2a). The superior margin of the N section was defined by the nasal base of the non-cleft side. The inferior boundary of the N section was defined by the root apex of the central incisor on the cleft side. The dental section was divided vertically in relation to the central incisor on the cleft side in an apical (A), mid apical (MA), mid coronal (MC) and a cervical (C) section (Fig. 2a). The A, MA, and MC sections were all equal in height. Due to an unequal number of slices, the C section differed with up to 0.5 mm (two slices) compared to the other dental sections.

Starting superiorly from the nasal base, every fifth axial slide was labeled to create a scaffold (Fig. 2b). The interpolation tool in ITK-SNAP was used to fill the segmentation in the slices in between the labels and generate a 3D object of the alveolar cleft. The volume of the alveolar cleft was calculated for the different sections and for the total cleft with the “volume and statics” function in ITK-SNAP. Erupting teeth with the surrounding dental follicle in the cleft zone was not counted as part of the cleft defect. Total initial cleft volume (VOLti) and dental initial cleft volume (VOLdi) of the alveolar cleft were calculated on the preoperative CBCT images. Total residual cleft volume (VOLtr) and dental residual cleft volume (VOLdr) were calculated on postoperative CBCT images. The bone graft outcome was calculated by comparing residual cleft volume with initial cleft volume giving the BF in ratio as a percentage according to the formula: (VOLi − VOLr)/VOLi × 100). A positive value refers to that the cleft decreases in size, while a negative value refers to that the cleft increases in size.

### Assessing the alveolar bone graft with 2D measurements

The same postoperative CBCT scans used for volumetric calculation were evaluated with two grading scales developed by Suomalainen *et al.* [6] and Liu *et al.* [4] to quantify the outcome of SABG by two of the authors (M.L, D.B).

For evaluation of the bone graft with the Suomalainen index [6], both axial and coronal slices of 0.25 mm were used. On the axial slices, the horizontal bone thickness was assessed on three vertical levels of the roots adjacent to the cleft. The horizontal bone thickness was classified into three categories dependent on the bone width in relation to the root width; poor, fair, and good with scores 1–3 according to the Suomalainen grading scale. Coronal slices were used to assess the vertical dimension of the bone graft. The vertical dimension of the bone graft was assessed in three vertical levels of the roots adjacent to the cleft; cervical, middle, and apical third of the root. The bone support was classified into poor, fair, or good with scores 1–3 dependent on how much the bone graft filled the given vertical section of the roots according to the Soumalainen grading scale. Coronal slices were also used to assess differences in height of the nasal floor on the cleft side compared to the non-cleft side Differences were classified into five categories, with scores 0–4. Score 0, indicating major differences, and 4, no difference in height of the nasal floor according to the Suomalainen grading scale. The sum of the horizontal, vertical, and the nasal scores was used as a cumulative score. The maximum cumulative score for the Suomalainen grading scale is 24.

On the same 0.25 mm axial slices used for the Suomalainen grading scale the bone grafts were assessed with the grading scale presented by Liu *et al.* [4] The interdental septal thickness in the bone-grafted alveolar cleft was assessed in relation to root width of the adjacent teeth. Assessments were performed at three vertical dental levels (cervical, middle, and apical third), with scores 1–4 according to the Liu grading scale. A lower Liu score indicated better bone support. According to the Liu grading scale, the worst outcome of the three vertical levels was used as the final result.

### Consensus on bone-grafted cleft outcome from a multidisciplinary meeting

Patient records regarding rehabilitation plan of the alveolar cleft were collected from a multidisciplinary meeting. At the meeting, the bone graft was judged as success (S) or regraft (R) based on the bone healing in the grafted cleft on CBCT scans according to the standard cleft treatment program.

### Statistical analysis

R Core Team (2021, version 4.1.1; R Foundation for Statistical Computing, Vienna, Austria) was used in the statistical analysis. The differences between the result groups for the outcome variables were assessed using different statistical tests. Mann–Whitney *U*-test was used for the Suomalainen grading scale. Independent *t*-test was employed for the volumetric outcome variables. Fisher’s exact test was used for the Liu grading scale. Correlation between the two grading scales were assessed by Spearman’s rank correlation. The level of statistical significance was set to 0.05.

### Intra- and inter-observer reliability

CBCT scans were analyzed by one orthodontist (M.L.) twice and one radiologist (D.B.) once. Intra- and inter-observer reliability tests were calculated for the volumetric measurements and analyzed with intra-class correlation (ICC). Cohen’s weighted kappa for intra- and inter-observer reliability were calculated for the Liu score. Intra-class correlation (ICC) values for intra- and inter-observer reliability were calculated for the Suomalainen cumulative score.

### Validity of the measurements

The grading scales and the volumetric measurements were compared in relation to the two outcomes, S or regraft R. If the result demonstrated a statistically significant difference between the two outcomes, the method was considered relevant and valid for evaluating a bone-grafted cleft. If the method demonstrated a lack of statistically significant difference between the two outcomes, the method was considered non relevant and not valid for evaluating the success of a bone-grafted cleft.

### Ethical approval

The Regional Ethical Review Board in Stockholm approved the present study (Dnr [daybook nr.] 2016/422-31 and Dnr 2019-04106).

## Results

### Bone graft outcome

The records from the interdisciplinary meeting showed that 15 (65%) of the bone-grafted alveolar clefts were judged as success and 8 (35%) of the bone-grafted alveolar clefts were judged as in need of a regraft. Data and statistics comparing group S and R are summarized in Table 1.

**Table 1.** Outcome variables related to result groups and sample mean.

	Success group (*n* = 15)	Regraft group (*n* = 8)	Sample mean (*n* = 23)	*P* value
*Total cleft*				
Initial cleft (mean mm^3^ (SD)) [min–max mm^3^]	699.8 (201.0) [434.7–1104.8]	759.0 (239.3) [474.3–1141.6]	720.4 (211.6) [434.7–1141.6]	0.536
Residual cleft (mean mm^3^ (SD))	211.1 (129.0) [39.6–459.5]	407.2 (140.9) [181.3–583.3]	279.3 (161.3) [39.6–583.3]	0.003
Bone fill (mean % (SD))	70 (15) [41.6–92.8]	45 (18) [15.8–65]	62 (20) [15.8–92.8]	0.001
*Dental cleft*				
Initial cleft (mean mm^3^ (SD))	414.2 (113.7) [199.5–592.5]	517.71 (175.9) [309.5–809.7]	450.2 (143.6) [199.5–809.7]	0.1
Residual cleft (mean mm^3^ (SD))	64.7 (51.8) [7.4–161.2]	241.90 (76.5) [118.2–314.8]	126.4 (105.0) [7.4–314.8]	<0.001
Bone fill (mean % (SD))	84 (13) [54.9–97.7]	51 (16) [31.9–75.9]	72 (21) [31.9–97.7]	<0.001
*Nasal cleft*				
Initial cleft (mm^3^)	285.7 (182.1) [68.1–556.9]	241.2 (113.7) [85.15–347.5]	270.2 (160.2) [68.1–556.9]	0.539
Residual cleft (mm^3^)	146.4 (132.0) [0–388.4]	165.3 (96.0) [18.0–268.5]	153.0 (118.7) [0–388.4]	0.726
Bone fill (mean %(SD))	47 (37) [−35.2 to 100]	19 (70) [−100 to 87.6]	37 (52) [−100 to 100]	0.220
	*n*	*n*	*n*	
Liu score 1 (%) 309.5	7 (46.7)	0 (0.0)	7(30.4)	0.052
Liu score 2 (%)	0 (0.0)	0 (0.0)		
Liu score 3 (%)	0 (0.0)	0 (0.0)		
Liu score 4 (%)	8 (53.3)	8 (100.0)	16(69.6)	
Suomalainen score (median (IQR))	19.0 (17.0, 21.0)	14.5 (13.25, 17.25)	18.0 (16.0, 20.0)	0.004

*P* values were calculated with a non-normative test.

### Volumetric bone graft outcome related to outcome groups

**Figure 3.**
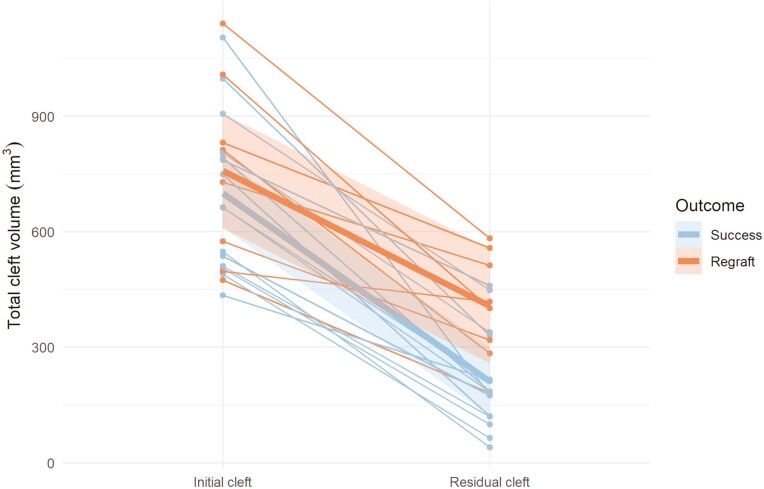
Changes of initial and residual total cleft volumes in mm^3^ for the success (*n* = 15) and the regraft (*n* = 8) groups. Individual changes (thin lines) and mean changes (thick lines).

**Figure 4.**
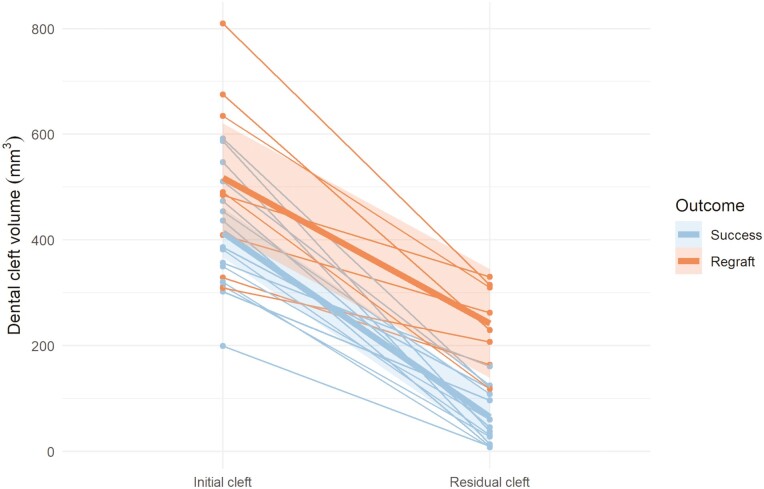
Changes of initial and residual dental cleft volumes in mm^3^ for the success (*n* = 15) and the regraft (*n* = 8) groups. Individual changes (thin lines) and mean changes (thick lines).

**Figure 5.**
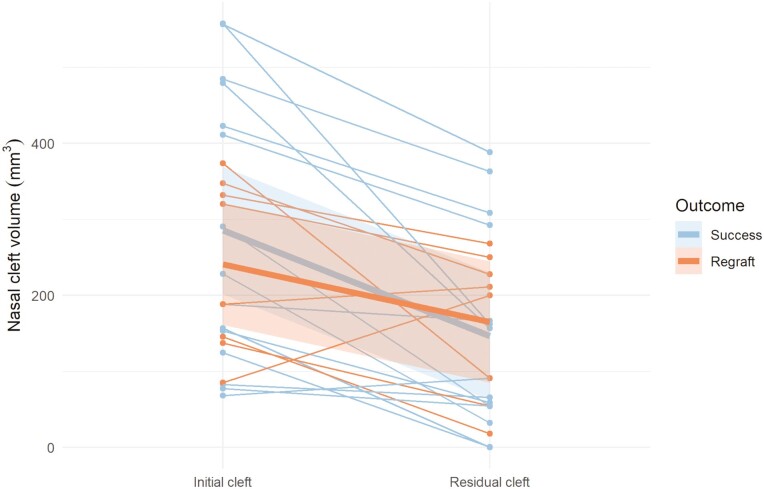
Changes of initial and residual nasal cleft volumes in mm^3^ for the success (*n* = 15) and the regraft (*n* = 8) groups. Individual changes (thin lines) and mean changes (thick lines).

**Figure 6.**
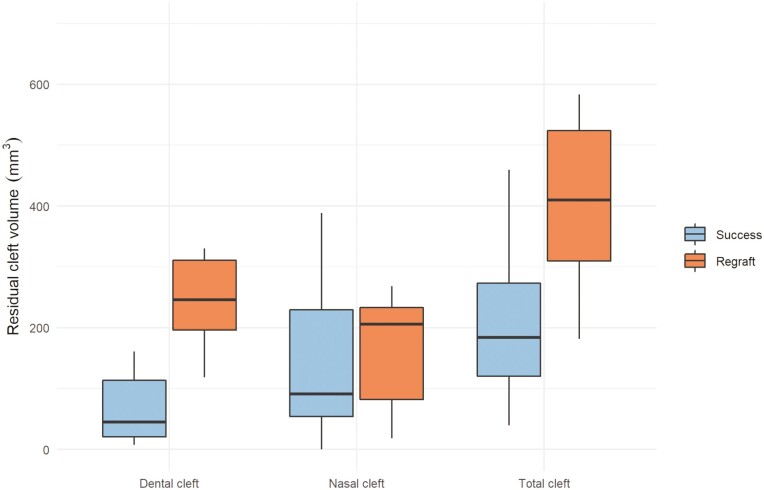
Dental, nasal, and total residual cleft volumes for the success and the regraft groups.

**Figure 7.**
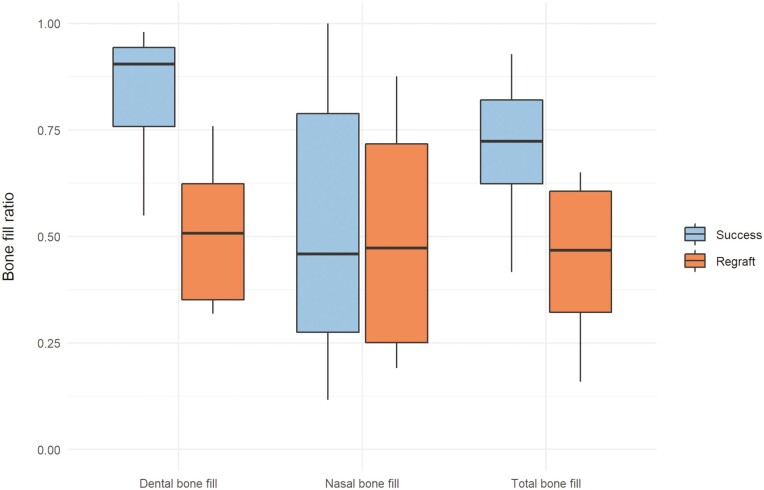
Dental, nasal, and total BF ratio for the success and regraft groups.

Individual changes and mean changes of the initial and the residual cleft volumes for the different outcome groups (S, R) are presented in Figs. 3–5. Residual cleft volumes are illustrated in Fig. 6. For the total and dental BF, a significant difference (*P* = 0.001, *P* < 0.001) was seen between the two outcome groups. However, no significant difference (*P* = 0.220) was seen between the outcome groups for the nasal BF. The BF ratio for the different outcome groups are presented in Fig. 7.

### Bone fill in different sections of the cleft and correlation to the grading scales

**Figure 8.**
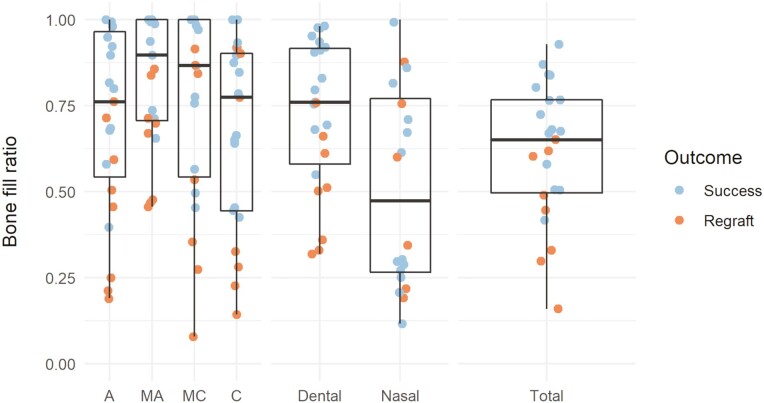
BF ratio for different sections of the cleft is divided into dental, nasal, and total. The dental section is further divided vertically in relation to the central incisor on the cleft side in an A, mid MA, MC, and a C section. The four dental sections were all equal in height.

The BF for the five different sections of the cleft is presented in Fig. 8. The BF in the dental parts was higher compared to the nasal part of the cleft in both groups. The highest BF ratio was seen in the two middle sections (MA, MC) of the dental section of the grafted cleft. The correlation between the dental residual cleft volume and the Suomalainen grading was −0.76 and with the Liu grading it was 0.70.

### The Suomalainen and the Liu grading scales related to outcome

The median cumulative score of the Suomalainen grading scale showed a significant difference (*P* = 0.004) between the outcome groups (Table 1). The mean cumulative score of the Suomalainen score was 17.52. The Liu grading scale did not show any significant difference (*P* = 0.052) between the outcome groups. All patients (*n* = 7) with Liu index 1 were from group S. The remaining patients (*n* = 16) were judged as Liu index 4, half of that group was from group S, and the other half was from group R. There was a strong negative correlation between the Suomalainen and Liu grading scales (−0.81).

### Intra- and inter-observer reliability

Intra- and inter-observer reliability test showed a strong agreement (ICC = 0.973, ICC = 0.952) for the VOLti. The intra- and inter-observer reliability test for the VOLtr also showed a strong agreement (ICC = 0.978, ICC = 0.950). The Liu score showed a strong agreement between ratings and raters (*κ* = 0.843, *κ* = 0.897). Intra-class correlation values for intra- and inter-observer reliability for Suomalainen cumulative score showed a strong agreement (ICC = 0.891, ICC = 0.837).

## Discussion

In the present study, the outcome after bone grafting the alveolar cleft was assessed on CBCT with 2D grading scales and 3D volumetric measurements. Results from 2D and 3D assessments were compared with each other and to the clinical reference (bone-grafted cleft outcome as defined by the multidisciplinary clinical consensus meeting) for validation. The anatomical boundaries of the alveolar cleft for the volumetric assessment were chosen in respect to clinical relevance and practicality. The nasal base on the non-cleft side was used as the superior boundary. Symmetry of the height of the nasal base is clinically relevant for the soft-tissue nasal shape. The cemento–enamel junction on the buccal surface on the cleft central incisor was chosen as a landmark, as it is easy to use and is in accordance with previous studies, Linderup *et al.* [21]. To define the palatal margin of the cleft, the incisive foramen was used as a landmark, as it is the posterior anatomical boundary of the alveolar cleft.

The volumetric assessment with a segmentation technique on CBCT images allowed for an evaluation with high reliability which is in accordance with other studies [5, 21, 22]. The mean total cleft defect in the present study was 720 mm^3^, which is in agreement with the study by Linderup *et al.* and Ellapakurti *et al.* [15, 21]. Other studies reported both lower and higher mean cleft defect volumes 629, 860, and 980 mm^3^ [5, 23, 24]. The reason for that could be how the boundaries are chosen.

Patients in the R group had higher initial cleft volumes than patients in group S. However, the difference was not significant. The BF ratio is calculated by comparing preoperative and postoperative cleft volumes. To note, is that the morphology of the maxilla changes due to growth and remodeling following bone grafting. In addition, teeth are erupting within the cleft zone. In the present study, the N section of the cleft increased in volume after bone grafting in three cases. The explanation for this is that the central incisor in the three cases were not fully erupted and thereby the N section increased in height when the central incisor erupted. The significant difference in residual volumes and BF ratio in the S and R group indicates that both methods of measuring is relevant for evaluating the bone-grafted alveolar cleft. This result raises the question if it is necessary to calculate the BF ratio which in addition to a postoperative CBCT also requires a preoperative CBCT examination. For planning surgery, it might be of importance to have preoperative CBCT, but calculating BF is probably not necessary for evaluation of the success of the bone graft for an individual patient. The BF ratio, on the other hand, might be a more appropriate method when evaluating a bone graft technique as the BF ratio shows how effective the intervention is to reduce the cleft volume. The point of dividing the cleft volume in different spatial locations was done to facilitate judgment of clinical favorable bone support at various levels. Bone support is essential for development of a healthy periodontium, vital to warrant further orthodontic tooth movements. Grading scales divide the cleft vertically in three dental sections to allow this distinction. Bone healing turned to be less successful in the nasal part than in the dental part. However, the nasal BF volume was not critical for the outcome at the multidisciplinary consensus meeting. Highest BF was seen in the middle part of the dental cleft, MA, and MC which might be the most critical sections for success of the bone graft.

The mean total BF ratio was 62%. This agrees with the study by Janssen *et al.* [25] but lower than the study by Lenderup *et al.* [21]. The lowest total BF ratio judged as success was 42%. The highest total BF ratio considered as need of a regraft was 65%. This result indicates the limitations of volumetric analysis for a final decision of a regraft or not. The decision for regraft or not should be supplemented with an individual analysis of every patient, based on dental status and future dental treatment plan.

The Liu grading scale [4] uses the minimum thickness at all levels as the final result. In the present study, 8 of the 15 patients from outcome group S were classified as Liu index 4. This result indicated that grading the minimum thickness at all levels might result in a poor outcome classification, even though regrafting is not needed. Yet, we need to consider that the Liu index was not developed with the intention to determine the need for cleft regrafting, but rather to assess the interdental septal thickness in the bone-grafted alveolar cleft.

In the report presented by Suomalainen *et al.* [6], the horizontal bone support in the cervical third of the root could only be assessed in 20% considering the artifacts from orthodontic appliances. This resulted in excluding the cervical horizontal measurements, while the present study could assess the cervical part. When excluding measurements from the horizontal cervical third, in the present study, the mean cumulative score of 15.4 was very similar to the mean cumulative score of 15.3 of Suomalainen *et al.* [6]. In the study presented by Liu *et al.* [4], the authors only evaluated CBCT images of patients that had been rated as successful on intraoral radiographs. This contrasts with the present study where data from consecutive patients were considered. A comparison between both studies is thus not appropriate as outcome parameters present some bias.

Both the Liu [4] and the Suomalainen grading scales [6] consider thickness and spatial location of the bone graft. The grading scales are faster to implement in the clinic than volumetric assessments, unless segmentation can be fully automated via artificial intelligence [26, 27]. The Liu grading scale was the fastest to perform (approximately 10–15 min), followed by the Suomalainen grading scale (around 20–25 min) and most time consuming was the volumetric measurements (ranging from 1.5 to 2 h). The correlation between the two grading scales and the dental residual cleft volume was strong. The two grading scales also correlate with each other. However, only the Suomalainen grading scale enabled to show a significant difference between S and R. The patient with the lowest Suomalainen score judged as success was 15, while 18 was the highest Suomalainen score for a patient needing a regraft. Comparable to volumetric analysis, results from grading scales seem to highlight the need for patient-specific analysis rather than simply relying on a calculated score to decide for regrafting. The sample size in the present study is limited due to the time frame that was set for the inclusion period. Further studies with increased sample size are, therefore, recommended in the future.

## Conclusions

Volumetric outcome assessment with a segmentation technique on CBCT images allowed for evaluation with high reliability and validity. Grading scales may be more efficient, as these do not require segmentation and may, therefore, be more clinically applicable than volumetric assessments. The validity of the Suomalainen grading scale was found good in contrast to the Liu grading scale, which tends to underestimate the bone graft outcome. For outcome assessment of the bone-grafted cleft it could thus be recommended using either volumetric measurements or the Suomalainen grading scale. Yet and at all times, a patient-specific analysis remains necessary when there is a need to regraft. The null hypothesis that there is no difference in outcome after bone grafting the alveolar cleft between the use of two different grading scales and 3D volumetric assessments was rejected in the studied population.

## Data Availability

The datasets analyzed in the current study are available from the corresponding author on reasonable request.

## Limitations

Due to the time frame that was set for the inclusion period the sample size is limited. The tube current and exposure time varied for the CBCT examinations. The reason for this was that different exposure protocols used during the time frame for the present study in the radiology department. However, the various protocols applied presented comparable image quality in terms of structure visibility and segmentation quality in the examination of the alveolar cleft [28].
