# Correction to: Validation and comparison of 2D grading scales and 3D volumetric measurements for outcome assessment of bone-grafted alveolar clefts in children

**DOI:** 10.1093/ejo/cjae062

**Published:** 2024-10-30

This is a correction to: Mathias Lemberger, Daniel Benchimol, Marie Pegelow, Reinhilde Jacobs, Agneta Karsten, Validation and comparison of 2D grading scales and 3D volumetric measurements for outcome assessment of bone-grafted alveolar clefts in children, *European Journal of Orthodontics*, Volume 46, Issue 2, April 2024, cjae002, https://doi.org/10.1093/ejo/cjae002

The originally published version of this manuscript has been amended to correct the following sentences, which had not been correctly deanonymized following peer review:

Since 2016, the cleft team in xx uses CBCT routinely to evaluate the bone-grafted alveolar cleft.

Consecutive records including CBCT scans from children born with a unilateral alveolar cleft who underwent reconstruction of the alveolar cleft with a bone graft from the iliac crest in xx were collected.

CBCT scans were analyzed by one orthodontist (x.x) twice and one radiologist (x.x) once.

The Regional Ethical Review Board in xx approved the present study (xxxxxxxxxx).

The affected sentences have been corrected to read as follows:

Since 2016, the cleft team in Stockholm uses CBCT routinely to evaluate the bone- grafted alveolar cleft.

Consecutive records including CBCT scans from children born with a unilateral alveolar cleft who underwent reconstruction of the alveolar cleft with a bone graft from the iliac crest at the Stockholm cleft team were collected.

CBCT scans were analyzed by one orthodontist (M.L.) twice and one radiologist (D.B.) once.

The Regional Ethical Review Board in Stockholm approved the present study (Dnr [daybook nr.] 2016/422-31 and Dnr 2019-04106).

